# Professional Dignity

**Published:** 1900-11

**Authors:** J. Morgan Howe

**Affiliations:** New York


### PROFESSIONAL DIGNITY.[1]

BY J. MORGAN HOWE, NEW YORK.

Mr. President and Gentlemen,—We have reached a high degree of discipline in New York, and disobedience to orders is something that no one dares consider, consequently I am here to bore you in answer to this toast. It reminds me of the story of Donald the Scotchman, who, in answer to an inquiry in regard to a new neighbor, what like of a man he was, said, "He is a curious laddie. I went down to have a bit talk wi' him the ither evenin', and he offered me a glass of whuskey. He was pouring it oot, and I told him to stop,—and he stoppit! That's the kind o' man he is." Obedience to orders sometimes makes those who issue them sorry, and I am sure that those who have issued these orders to me are sorry now, or will be. But I promise you, Mr. President and gentlemen of New England, I will consider your defenceless situation at this late hour.

It is long since the immortal dramatist put into the mouth of Aragon, in the "Merchant of Venice," "Let none presume to wear an undeserved dignity;" and probably ever since that time there has been some danger that dignity would be assumed undeservedly. As civilization advances and becomes more complex its outgrowths assume the conditions and principles that underlie them, and I hold that the principles pertaining to a profession are very similar to the fundamentals of civilization itself. We remember that Emerson said, in his "Essay on Civilization," that "steam, electricity, rapid-fire guns, gum shoes, and such like evidences of intellect, were the toys thrown off in the exuberance and exhilaration of the security and freedom of a healthy morality in society." And he said also that morality was the one essential in civilization. You, gentlemen, who hear me do not need any suggestions in regard to morals, professional ethics, or anything of that kind, but there is not one of us who is not lowered in our status by the methods of advertising that are employed in the different cities of this country. The dark-skinned drummers in uniform inviting victims into den-

---

[1] Remarks made at the Union Meeting of The New York Institute of Stomatology and the American Academy of Dental Science, Boston, March 21, 1900. Its reception was delayed, and hence appears unavoidably at this late date.—Ed.

tal parlors, vying with dental departments in department stores, are inconsistent with dignity, but almost all these institutions are conducted by graduates of dental schools. It seems to show that the dental colleges of the land do not succeed in inspiring their graduates with an ambition to be more than tradesmen. The desire for material gain entirely supersedes the wish to be worthy members of a profession. We look entirely to colleges, to journals, to societies, and to laws for the means of professional elevation. I hold that no matter how learned the members of our craft may be, no matter how skilful, if their desires and aspirations are on the level of a factory, and they are willing to accept a strictly business relation to their *confrères* and the public, they are not elevating the profession, but lowering its status and detracting from its dignity. It has been reported, as a private opinion of Kipling's, that Cecil Rhodes needed no morals,—he was building an empire. I would set over against that remark an expression made by President Eliot (whom we have had the honor of claiming as a guest to-night) in an after-dinner speech in New York not long ago, when he said that the past year had been the most prosperous that Harvard had ever enjoyed, and he added, in explanation, that he meant by prosperity that the intellectual and spiritual element of the University had grown in power and strength. I think that remark is in key with our claim, that the status of educational institutions, and of professions that depend upon them for the equipment of their members, cannot be expressed in terms of a mill or factory, but in those of intellectual and moral relations. Yet our dental journals are constantly referring to what they call "professional progress" in terms that are applicable only to materials and methods.

It seems to me that there is much to be desired in ethical progress, or its equivalent, professional progress. There is no doubt in this age that scientific progress will take care of itself. The tide of material advancement of our time will carry us along whether we will or no, but moral progress demands that we should resist the current, in which we seem to be beyond our depth; in which we have lost our footing. It seems to me that acquiescence in the influence exerted upon our moral sentiments through trade channels, and the control of our literature by corporations that are supplying us with goods, is inconsistent with true dignity. We are indebted to manufacturers for enterprise in furnishing supplies, but we ought to cut ourselves loose from them more than we have in the matter of ethics. We need, in the first place, to decline to continue to use secret preparations. Almost all of us are using preparations of which we know nothing of the composition except in a general way, and many of the colleges, if not all of them, are teaching the students to use alloys that they know only by the name of the maker. Practitioners of dentistry constitute the majority of the stockholders in a supply company that urges the sale of its secret compounds in advertisements that claim that *our make* is the only one fit to use. The corporation owns and sells patent devices, while denouncing indefinitely the abuses of patents in the hands of others. And the president of the company is an officer of the National Association. Certainly these things do not suggest professional dignity.

The commercial control of our literature is demoralizing. We may say that it amounts to nothing; that the instances are very few or unknown in which there has been any interference with a free expression of opinion. Yet the ownership and control are for commercial purposes, and they are supreme. These patent conditions, in which we acquiesce, are going on from year to year. They might be rectified by a united effort; and decision on the part of just such societies as we are would be potent to accomplish a great deal in elevating the moral tone of societies, colleges, and practitioners. In proportion as this is accomplished will we deserve a dignified position in the community.

